# Cognitive testing of an instrument to evaluate acceptability and use of pre‐exposure prophylaxis products among women

**DOI:** 10.1002/acp.3590

**Published:** 2019-07-23

**Authors:** Seth Zissette, Millicent Atujuna, Elizabeth E. Tolley, Eunice Okumu, Judith D. Auerbach, Sally L. Hodder, Sevgi O. Aral, Adaora A. Adimora

**Affiliations:** ^1^ Behavioral, Epidemiological & Clinical Sciences FHI 360 Durham USA; ^2^ Desmond Tutu HIV Centre University of Cape Town Cape Town South Africa; ^3^ Department of Medicine University of California San Francisco School of Medicine San Francisco USA; ^4^ Section of Infectious Diseases West Virginia University School of Medicine Morgantown USA; ^5^ Division of Sexually Transmitted Disease Prevention Centers for Disease Control Atlanta USA; ^6^ Department of Epidemiology University of North Carolina at Chapel Hill Chapel Hill USA

**Keywords:** acceptability, clinical trials, cognitive interviewing, HIV prevention, PrEP

## Abstract

Given the range of pre‐exposure prophylaxis (PrEP) products currently being tested to prevent HIV in women, a standardized Acceptability and Use of PrEP Products Among Women Tool may facilitate comparisons of product acceptability and use across different geographies, trials, and users. We conducted three rounds of cognitive interviewing over 2 months in 2016, with 28 South African women who had experience participating in a range of PrEP product trials. The final instrument contained 41 items, including five new items that improved construct validity and 22 items modified for clarity. Changes were made due to unclear wording, difficulty answering, participant embarrassment, low response variability, and administrative formatting. Cognitive interviewing provided a means to address issues that would have inhibited this tool's ability to accurately collect data otherwise. This rapid, low‐cost study provided valuable insight into participants' understanding of questions and demonstrated the utility of cognitive interviewing in international clinical trials.

## 1 INTRODUCTION

Studies in recent years have demonstrated the effectiveness of biomedical approaches to HIV prevention, especially the use of pre‐exposure prophylaxis (PrEP) products (such as oral pills, vaginal gels, and vaginal rings) that use antiretrovirals to reduce HIV transmission risk for uninfected individuals (Cáceres et al., 2015). According to the AIDS Vaccine Advocacy Coalition as of December 2016, 55 PrEP open label, demonstration, and implementation projects were ongoing or planned globally in 33 different countries, over half (17) in sub‐Saharan Africa. Of these, more than one third (20) focus primarily or exclusively on women (AIDS Vaccine Advocacy Coalition (AVAC), 2016). Women's preferences and needs for PrEP products have been shown to differ across settings (Mack et al., 2014; Severy, Tolley, Woodsong, & Guest, 2005; Tolley et al., 2010, 2014; Tolley & Severy, 2006). Understanding how these preferences for and experiences using different PrEP products vary is critical.

Characteristics of trial participants, the clinical trial setting in which participants find themselves, and types of products can influence product acceptability and adherence (E. Tolley et al., 2014; Amico & Stirratt, 2014; Mack et al., 2014). Partner context and perceptions of risk can influence whether products will be acceptable within the relationship or need to be used, respectively (Tolley et al., 2014). Previous work has also demonstrated conflicts between communities and clinical trials themselves that may affect product uptake (Amico & Stirratt, 2014; Saethre & Stadler, 2013; Tolley et al., 2014). Additionally, long‐acting injectable PrEP in particular can present vastly different acceptability and adherence issues from more “on‐demand” frequent self‐use products like oral PrEP, vaginal gels, and vaginal rings (Landovitz, Kofron, & McCauley, 2016; Mastro, Sista, & Abdool‐Karim, 2014; Tolley et al., 2014).

Given the range of products and issues, a standardized tool may facilitate comparisons of product acceptability and use across different geographies, trials, and users. In this study, we provide initial validation for the Acceptability and Use of PrEP Products Among Women Tool, a standardized instrument that can be used to assess product use and trial experience of women in PrEP product clinical trials. The instrument is intended for use in oral, vaginal ring, vaginal gel, and long‐acting injectable PrEP trials.

We conducted cognitive interviewing, a technique that employs both structured and open‐ended questions, as a means of assessing the draft tool. Arising out of Cognitive Aspects of Survey Methodology (CASM), a field that emerged in the 1980s as a framework to understand the cognitive sources of survey response error (Willis, 2004), the goal of cognitive interviewing is to obtain information about survey questions in order to assess their quality and construct validity (Beatty & Willis, 2007). The process provides advantages over traditional pilot testing by both identifying problems in survey design and the cause of such problems, as well as assessing comprehension, information retrieval, and response quality of questions (Collins, 2003). Cognitive interviews differ from qualitative interviews in that qualitative interviews focus on individuals' experiences and opinions about events in their lives, whereas cognitive interviews focus much more narrowly on the thought patterns used by individuals to respond to questions. The answers to the questions in the cognitive interview guide are not intended as the primary results themselves; rather, the primary results are the participants' understandings of those questions and suggestions of how to clarify the questions. In more recent years, cognitive interviewing has been increasingly used to ensure comparability of survey items across cultural and linguistic groups (Willis & Miller, 2011).

## 2 METHODS

### 2.1 Creation of the initial instrument

The HIV Prevention Trial Network (HPTN) Women at Risk (WAR) Committee was tasked with developing a brief standardized tool to assess women's experiences with investigational products that could be used across different trials. The draft tool was developed by drawing on questions that mapped onto an acceptability framework (as described earlier) and had been used in previous acceptability studies of HIV prevention products (i.e., oral PrEP and microbicides) in India, eastern Africa, and the United States (Guffey et al., 2014; Hodder et al., 2013; Mehendale, Deshpande, Kohli, Tsui, & Tolley, 2012). An initial tool was reviewed and revised in consultation with the WAR committee prior to initiating the cognitive interview process.

### 2.2 Setting

In November and December 2016, we conducted cognitive interviews in an on‐going injectable PrEP research site (the Desmond Tutu HIV Foundation in Cape Town, South Africa). We focused cognitive testing of this tool among women who had recently participated in a long‐acting injectable trial as there is less experience with acceptability assessments of injectables compared with previous “on‐demand” products (e.g., oral). At the same time, the site also had access to women who had participated in other PrEP product trials, allowing us to simultaneously examine use of the tool for multiple different product types.

### 2.3 Data collection

Three rounds of cognitive interviews were conducted with a minimum of six interviews per round. We administered the cognitive interviews with former HPTN and Microbicide Trials Network (MTN) trial participants, purposively selected to represent a range of sociodemographic characteristics (“young” women, defined as ages 18–24; and “older” women, ages 25 and older) and HIV prevention products (injectable PrEP, oral PrEP, and vaginal ring PrEP). For each stratification of age and product, we aimed for inclusion of approximately three women per round. Spoken fluency in either English or isiXhosa was required for study participation (literacy in either was not required), but we prioritized selecting participants with comprehension of both languages.

Interviews were audio‐recorded to allow interviewers to write detailed summary notes after each cognitive interview. For each interview, the interviewer read aloud each item and its response options to the participant, and the participant orally responded. Each interview was conducted in a mix of both English and isiXhosa, with the interviewer reading both options to the participant to assess her understanding of each version and to allow her to respond in either language as desired. Interviews were only summarized in English. Interviewers completed data extraction sheets that summarized participants' responses, behavioral reactions, difficulties with the guide, concerns, and suggestions. The draft questionnaire was divided into eight sections—demographics, general risk behaviors, partner context(s), HIV risk perception, risk reduction behaviors, study product use, acceptability, and clinical trial participation. The initial draft contained 36 questions, with 11 alternate phrasings added for testing and probes for interviewers added throughout.

### 2.4 Data analysis

Analyses were based primarily on the data extraction sheets and focused on the items in the questionnaire. At the end of each round, the full study team met via telephone to identify any questions or items that appeared to show low or no variability in responses, and that were identified as unclear, embarrassing, difficult to respond to, or lacking relevance. These items were modified to produce a revised set of acceptability questions and tested in the next round until the team was satisfied that all options had been sufficiently tested. In addition to participant feedback, interviewer feedback was also incorporated to adjust framing, skip pattern usage, and flow of the questionnaire.

### 2.5 Ethical considerations

Ethical approval for this project was obtained from the ethical review boards at FHI 360's Protection of Human Subjects Committee and the University of Cape Town's Human Research Ethics Committee (Ref #: 640/2016). Written consent was obtained from each participant both to conduct and audio‐record each interview. Data were de‐identified to protect participant confidentiality.

## 3 RESULTS

### 3.1 Sociodemographic information

The cognitive interview sample included 28 women who had previously participated in an HIV prevention product clinical trial (Table 1). Most participants (60. 7%) were young women, and most of the overall sample had either participated in an injectable PrEP trial or vaginal ring trial (46. 4% and 39. 3%, respectively). Within the two age strata, most young women (52.9%) were injectable PrEP trial participants, and most older women (54. 5%) were vaginal ring trial participants.

**Table 1:** Cognitive interview participant sociodemographic characteristics

Characteristic	Young women, ages 1–24 (*n* = 17)	Older women, ages 25+ (*n* = 11)	Total (*n* = 28)
Age			
Mean (years)	22.6	33.2	26.8
Range (years)	19−24	25−44	19−44
Proportion of total (%)	60.7	39.3	100
Trial type (%)			
Injectable PrEP	52.9	36.4	46.4
Oral PrEP	17.6	9.1	14.3
Vaginal ring PrEP	29.4	54.5	39.3

### 3.2 Changes to questionnaire

The final instrument (Appendix 1) contained 41 items, including five new items that improved construct validity; 22 items were modified for clarity. Changes to the draft questionnaire were made iteratively across three rounds of data collection. Questions and response options were added or modified based on participant comprehension of the question and response format, embarrassment caused by the question, and variability of responses generated by the question. Formatting adjustments were also made based on feedback from interviewers on usability of the questionnaire. Twenty‐eight content‐based changes and 16 administrative/formatting changes were made. Changes are summarized in Table 2.

**Table 2:** Summary of changes to questionnaire

Reason for change	Number of changes
Content‐based changes	
Unclear wording	
Question	7
Response options	8
Difficult to answer	
Question	4
Responses	4
Embarrassment	4
Response variability	1
Administrative changes	
Formatting	16

#### 3.2.1 Changes due to unclear wording

Most content‐based changes were made due to unclear wording in questions or response options. These were questions that used vocabulary or complex phrases unfamiliar to the participants, as well as questions that were difficult to translate into isiXhosa or for which the isiXhosa translation differed greatly from the intended English version. To resolve these issues, interviewers worked with participants to uncover the meaning behind the question or response option, then find phrasing within the participants' vocabularies that applied to the question or response option without changing its meaning. Questions with vocabulary that could be interpreted variably by different participants are also included in the category; for instance, participant definitions of the differences between “rarely,” “sometimes,” and “frequently” varied greatly and likely did not capture experiences in the same way. Examples of questions changed due to unclear wording are presented in Table 3.

**Table 3:** Examples of changes made due to unclear wording

Original item	Change made	Reason for change
“Did you ever feel stigmatized because you were using (this product)?”	“Did you ever feel that people looked at you differently because you were using (this product)?”	Participants struggled to understand the word “stigmatized” in both English and isiXhosa. Interviewers suggested this would convey the meaning of the question in a more easily understood phrasing. Subsequent testing with participants indicated that their definitions of “looked at you differently” fell closely in line with our definition of “stigmatized.”
“At your most recent sexual encounter, did you discuss condom use with your sexual partner?”	“The last time you had sex, did you discuss condom use with your sexual partner?”	The phrase “At your most recent sexual encounter …” confused participants in English. When interviewers translated the question and when participants were asked how they would translate this question, the isiXhosa phrasing translated more closely to “The last time you had sex ….” When tested in English, participants did not experience the same problems they did with the original phrasing.
“Since your last visit, how often did you use the (study product)? ‐ Never ‐ Rarely ‐ Sometimes ‐ Frequently ‐ Always”	“Since your last visit, how often did you use the (study product)? ‐ Never ‐ Less than half the time ‐ More than half the time ‐ Always”	Participants struggled to define and differentiate between (and often had differing definitions of) “rarely,” “sometimes,” and “frequently,” with “sometimes” particularly providing trouble for participants and being difficult to translate for interviewers. “Less than half the time” and “More than half the time” were more intuitive phrases in both English and isiXhosa, and when asked in tandem with a question about the time passed since their last study visit, provided a more accurate picture of product use.

#### 3.2.2 Changes due to difficulty answering

Following changes due to unclear wording, most other content‐based changes were made because the questions were difficult to answer. The difference between unclear wording and difficulty answering was one of construct validity; questions with unclear wording had construct validity as long as the participants had the vocabulary necessary to understand the question, whereas questions that were difficult to answer were either asked in a way—or had response options—that did not accurately reflect the experiences of participants. For instance, some multiple‐choice questions about non‐use of the study product lacked an answer choice that reflected the participant's reason for not using the product. Similarly, some questions asked participants to recall information they could not. Examples of questions changed due to difficulty answering are presented in Table 4.

**Table 4:** Examples of changes made due to difficulty answering

Original item	Change made	Reason for change
“Since your last visit, how many times did you use the (study product)?”	“Since your last visit, how often did you use the (study product)?” ‐ Never ‐ Less than half the time ‐ More than half the time ‐ Always”	Participants who used on‐demand products experienced difficulty recalling the number of times they used the product, especially if the time between visits was more than 2 weeks. Participants were only accurately able to provide a more general sense of their product use and suggested the change in wording and a Likert response scale to moderate the accuracy of the question.
“The last time you had sex, how willing were you to have sex? Would you say you … … ‐ Had sex for money or drugs”	“The last time you had sex, how willing were you to have sex? Would you say you … … ‐ Had sex for material things, money or drugs”	Other response options focused on willingness to have sex (i.e., willing, coerced, or forced); this was the primary option for capturing transactional sex. Participants and interviewers thought that it did not accurately reflect instances they had heard of transactional sex in this community, usually had for material gifts from “blessers.” Participants thought that adjusting the wording would capture more accurate responses.
“Participants may not always use their (study product) as directed for many reasons. I will read a list of possible reasons some participants may have missed (taking a pill/applying their gel/using the vaginal ring). Were any of the following a reason why you DID NOT use the (study product)? Note: Read all responses aloud. Mark all that apply.”	Added answer choice: “‐ Worried partner would feel product during sex”	Eleven different response options representing common reasons for missing product use (e.g., being away from home without product, worrying about side effects of product, and running out of product) were presented. However, participants who had used vaginal gel or vaginal ring PrEP repeatedly indicated that worry their partner would feel the product during sex was a major concern for them and not listed. Adding this as a response option led to it being selected by many participants, therefore providing a more accurate picture of issues around product use.

#### 3.2.3 Changes due to embarrassment

Anal sex was so highly stigmatized among participants that even when asked about it in conjunction with vaginal sex, women who had only had vaginal sex were uncomfortable answering the question. Participants experienced strong feelings of embarrassment about such questions as, “How would you describe the last man you had vaginal/anal sex with?”—focusing in on the anal sex component of the question. Participants who had previously had anal sex suggested that they would not mind answering questions about anal sex separately from vaginal sex, so questions relating to vaginal and anal sex were split to decrease embarrassment. Following the split of questions into separate ones about vaginal and anal sex, participants responding to questions about vaginal sex no longer experienced extreme embarrassment, and participants who had had anal sex reported no problems answering the separate questions.

#### 3.2.4 Changes due to low response variability

One question was changed due to low variability in responses from participants. When responding to the question, “How easy is the [study product] to use?” all participants selected the response “Very easy.” The interviewers hypothesized that this could be a socially desirable response to the wording of the question and decided to test the alternate phrasing, “How difficult is the study product to use?” which did generate a wider range of responses in subsequent rounds.

#### 3.2.5 Administrative changes

Administrative changes were largely those suggested by the interviewers themselves to improve their ability to use the questionnaire. These included the addition of framing before each section to prompt participants toward the section's subject matter and splitting long, complex questions about multiple concepts into separate shorter questions for simplicity. The use of skip patterns for relevant questions based on the answers of previous questions was also suggested by not only interviewers but also participants, who grew frustrated by questions that did not apply to their case and could easily be determined from previous answers.

## 4 DISCUSSION

Our study identified issues in many questions that would have inhibited the questionnaire's ability to accurately collect data in this setting. Cognitive interviewing provided a pathway to preemptively and comprehensively address these issues to improve the questionnaire. The problems identified in our study were similar to those found by Carrasco (2003), who identified four types of linguistic issues and three types of design issues. Linguistic issues were: (a) pre‐existing cultural definitions, including cultural meaning of intended phrases not consistent with the intended meaning (e.g., local colloquial names of institutions); (b) having no translational equivalent marker for the English phrase (e.g., concepts that exist in one culture but not another); (c) determining the most frequently occurring vocabulary for terms with multiple translations (e.g., for words with many synonymous translations, determining the one that will be most familiar to participants); and (d) literal translations that did not map onto the original English term (e.g., idioms). Design issues were: (a) correctly mapping responses onto discrete response choices (e.g., providing response options that will make sense to participants who are asked the question), (b) effects on later questions triggered by question order (e.g., negative connotations of a word in one question affecting the interpretation of later questions), and (c) errors due to automated text (e.g., different grammar structures between languages; Carrasco, 2003).

In our study, revisions were made due to unclear wording (Table 3), which could be attributed to difficulty determining the translation equivalent for questions and responses and to difficulty determining the most frequently occurring vocabulary for terms. Similarly, revisions were also made because of difficulty responding to the question, which usually arose due to problems mapping responses onto the correct choice, including the complete absence of a correct choice. Pre‐existing cultural definitions could also help to explain the strong embarrassment associated with questions about anal sex. Some of our other revisions were more distinct from the types of changes outlined by Carrasco. Determining the most frequently occurring vocabulary included not just the English‐to‐isiXhosa translational vocabulary, but also converting the American English terminology to South African English. While making these changes, we also had to consider that in addition to intra‐language dialect differences, South Africa alone has 11 official languages and multiple different language families. A high level of linguistic precision for items was needed to ensure that we could begin adapting items to the South Africa context, which will aid in future efforts to use them in other southern African settings. In addition to the linguistic issues outlined above, low response variability for some questionnaire items prompted revisions to increase variability and enhance data quality.

The clinical trial setting creates tensions between the need to ask participants the right questions to gauge product acceptability and use and the need to avoid overburdening them with lengthy questionnaires in addition to other trial demands. We believe the cognitive interview process yielded questions that participants are more likely to understand and accurately answer. We furthermore believe that this approach improves upon the usual development of questionnaires for clinical trials by addressing participants' understanding of questions and decreasing the uncertainty about the comprehensiveness of the instrument and its ability to be used across different sites and products. Cognitive interviewing can be done relatively quickly during the course of most clinical trials for HIV prevention products.

The major limitation of this study is the geographic and cultural constraint imposed by conducting it in only one site. The linguistic precision and cultural adaptation needed to improve items serve as initial work in adapting questions to a context outside of that for which they were originally created. We originally intended to conduct the study in two countries and translate the instrument to two languages, but timing and budgetary constraints necessitated limiting the work to South Africa. One issue that contributed to our inability to conduct the study in a second site may have been the limited use of cognitive interviews in developing questionnaires for past clinical trials. Because the aim of our cognitive interviewing process was to improve study instruments rather than assess participant attitudes and behaviors *per se*, the process potentially could have been considered “non‐research,” or could have received an expedited review by institutional review boards (IRB). Nevertheless, local IRB reviews in both countries processed the protocol as it might any qualitative sub‐study to a clinical trial, requiring multiple steps and ultimately preventing us from carrying out the work in a second country. Increased visibility of cognitive interviewing benefits in the clinical trial context and the development of more expedited review processes could help to overcome the method's lack of inclusion in clinical trials.

This rapid, low‐cost cognitive interview study provided valuable insight into participants' understanding of PrEP acceptability questions, enabling us to further refine the instrument for use in the South African setting. In addition, this study has shown the utility of cognitive interviewing in international clinical trials. Both the initial refinement in a southern African setting and the demonstration of the usefulness of cognitive interviewing will facilitate adaptation of the questionnaire for settings beyond South Africa. Cognitive interviewing and the resultant revisions of the instrument have increased confidence in the validity of the questions, framing, and responses, as well as the ability of the instrument to capture multiple components of product acceptability and use. Further validation of the instrument in other geographic settings will improve our ability to examine and compare women's preferences and needs for PrEP across multiple contexts. Similarly, the improvement in validity offered by cognitive interviewing will outweigh any complexity added by its inclusion in future trials, which will ultimately affect both efficacy and effectiveness of products. The outcomes of women‐focused PrEP trials to date demonstrate the value of the cognitive interviewing process.

## CONFLICT OF INTEREST

The authors have no conflict of interest to declare.

## Supporting information


**Data S1.** ACCEPTABILITY AND USE OF PREP PRODUCTS AMONG WOMEN TOOL
