# Supplementary material for: Cognitive testing of an instrument to evaluate acceptability and use of pre‐exposure prophylaxis products among women
Source: Appl Cogn Psychol. 2019 Jul 23;34(1):78–84. doi: 10.1002/acp.3590 (PMC7003830; doi:10.1002/acp.3590)
Supplement: Supplementary file 1 — Data S1. ACCEPTABILITY AND USE OF PREP PRODUCTS AMONG WOMEN TOOL [file ACP-34-78-s001.docx]

**ACCEPTABILITY AND USE OF PREP PRODUCTS AMONG WOMEN TOOL**

Instructions

Orientation to Survey:

This survey was developed in collaboration with the Women at Risk (WAR) Committee which was tasked with developing a brief standardized tool that can be used across different HIV prevention clinical trials to assess women’s experiences with investigational products.

The survey instructions, questions and response options were tested, using a cognitive interviewing approach, among former participants of several different clinical trials in Cape Town, South Africa. (See report 2017 for further information.) The instrument has not been tested in other geographic settings. Therefore, it may be useful to pretest the instrument prior to using in another geographic/linguistic context.

The survey contains 8 sections. It may be used as a stand-alone survey or as a complement to other survey instruments and administered either cross-sectionally or longitudinally. Some sections require tailoring to the product use context of the clinical trial. Considerations are described below:

1. DEMOGRAPHICS
   1. For clinical trial research within HPTN or other NIH-funded networks, the standard socio-demographic questions which correspond to DAIDS economic and social (ES) categories should be used in place of questions 1.1 through 1.4.
   2. When conducting auxiliary or stand-alone studies in geographic settings other than South Africa, questions 1.3 and 1.4 will require adaptation to ensure they fit the local context.
2. GENERAL RISK BEHAVIORS
   1. The interviewer should note if responses to 2.2 or 2.3=1. Additional questions should be administered in section 5 on Risk Reduction Behaviors.
3. PARTNER CONTEXT(S)
   1. Participants should choose only one response for question 3.3.
   2. Questions 3.4 and 3.5 only apply to participants who report being married or having regular partner(s).
   3. Question 3.4 implies that a participant spends more time living together with her partner than living apart.
   4. Question 3.5 implies that a partner routinely resides in a different town for work. It does not apply to work-related travel that may be infrequent or short-term (i.e., 1-2 weeks).
4. HIV RISK PERCEPTION
   1. This section asks about generalized feelings of HIV risk, as well as perceived risk from own or partner behaviors.
5. RISK REDUCTION BEHAVIOURS
   1. Follow-up visit schedules may vary for different clinical trials; some may require monthly visits, while others require quarterly visits. And, participants may miss visits or come for unscheduled visits. Question 5.1 aims to document the last scheduled or unscheduled visit that a participant made.
   2. The timeframe used for Questions 5.2 and 5.3 should be determined based on follow-up schedule. A timeframe of 3 months is suggested for HPTN studies. Auxiliary studies, or studies that have different follow-up schedules may choose to shorten timeframe (i.e, in the last month) or reference the timeframe provided in question 5.1.
   3. Note that the response options for question 5.2 have been tested with a general population of South African women. If a study is being conducted in a population with much higher frequency of sexual acts, then it is advisable to record the number of sexual acts, rather than to record a category from the range of responses provided.
6. STUDY PRODUCT USE
   1. This section assesses product use for a range of products that are under the participants’ control. It does not apply to products, like an injectable PrEP product, that must be administered by a healthcare provider.
   2. Some questions require the interviewer to replace [study product] with the specific type of product being evaluated within the clinical trial.
   3. Administer only the questions that are applicable to the specific product(s) being used within the clinical trial.
   4. Question 6.4 may be asked in any HIV prevention clinical trial, regardless of whether the investigational product is under provider or participant control.
7. ACCEPTABILITY
   1. Some questions require the interviewer to replace [study product] with the specific type of product being evaluated within the clinical trial.
   2. In questions 7.2-7.4, note that some questions apply only to specific product(s).
   3. It may be helpful to develop a set of visual cards – one for each product – with a brief description of how it is used and how it works. Participants could then briefly look at cards before responding to question 7.6.
8. CLINICAL TRIAL PARTICIPATION
   1. We recommend that questions 8.1 through 8.6 be asked only at the final study visit (or the last visit when products were used.)
   2. In question 8.2, the intent of “people looked at you different” is in a disapproving way.

| **ACCEPTABILITY AND USE OF PREP PRODUCTS AMONG WOMEN TOOL** | | |
| --- | --- | --- |
| Version 3.0 | | |
|  |  |  |
| **1. DEMOGRAPHICS** | | |
| Thank you for taking the time to meet with me today to share your thoughts about this study product and clinical trial with me. I would like to first ask you some questions about yourself and your situation. | | |
|  |  |  |
| 1.1. | How old are you? | \|___\|___\| |
|  |  |  |
| 1.2. | What is your highest level of education? | \|___\| |
|  | 1 = No schooling |  |
|  | 2 = Primary school, not complete |  |
|  | 3 = Primary school, complete |  |
|  | 4 = Secondary/high school, not complete |  |
|  | 5 = Secondary/high school, complete |  |
|  | 6 = Attended college or university, not complete |  |
|  | 7 = Attended college or university, complete |  |
|  |  |  |
| 1.3. | What is your race? | \|___\| |
|  | 1 = African / Black African |  |
|  | 2 = European / White |  |
|  | 3 = Indian / Asian |  |
|  | 4 = Mixed Race / coloured |  |
|  | 5 = Other |  |
|  |  |  |
| 1.4. | What is your income status? | \|___\| |
|  | 1 = No income |  |
|  | 2 = Government grant |  |
|  | 3 = Income from full-time employment |  |
|  | 4 = Income from part-time employment |  |
|  | 5 = Income from self-employment |  |
|  | 6 = Other, specify: _________________________________________________ |  |
|  |  |  |
| **2. GENERAL RISK BEHAVIORS** | | |
| Now I would like to ask you some questions about general risk behaviour. Some of these questions may be embarrassing to answer. | | |
|  |  |  |
| 2.1. | Have you had vaginal sex in the last 3 months? | \|___\| |
|  | *Note: Use a calendar or other reference to clarify 3 months prior from today's date.* |  |
|  | 0 = No |  |
|  | 1 = Yes |  |
|  |  |  |
|  |  |  |
|  |  |  |
| 2.2. | Have you had anal sex in the last 3 months? | \|___\| |
|  | 0 = No |  |
|  | 1 = Yes *[SKIP TO 3.1.]* |  |
|  |  |  |
|  | *[IF 2.2 = 0]* |  |
| 2.3. | Have you ever had anal sex? | \|___\| |
|  | 0 = No |  |
|  | 1 = Yes |  |
|  |  |  |
| **3. PARTNER CONTEXT(S)** | | |
| Next we will talk about your partner context. | | |
|  |  |  |
| 3.1. | How many partners have you had vaginal sex with in the last 3 months? | \|___\|___\| |
|  | *[IF 0, SKIP TO 3.3.]* |  |
|  |  |  |
| 3.2. | Of these, how many were new sex partners? | \|___\|___\| |
|  |  |  |
| 3.3. | How would you describe your current relationship status? Would you say you are… | \|___\| |
|  | *Note: Read all options. Mark the response that best describes the participant’s current relationship status.* |  |
|  | 1 = Married |  |
|  | 2 = Have regular partner(s) |  |
|  | 3 = Sexually active, but no regular partner(s) *[SKIP TO 4.1.]* |  |
|  | 4 = Not sexually active currently *[SKIP TO 4.1.]* |  |
|  |  |  |
|  | *[IF 3.3 = 1 OR 2]* |  |
| 3.4. | Do you live together most of the time? | \|___\| |
|  | 0 = No |  |
|  | 1 = Yes |  |
|  |  |  |
|  | *[IF 3.3 = 1 OR 2]* |  |
| 3.5. | Does your husband or regular partner work in another city that requires him to live away from home sometimes? | \|___\| |
|  | 0 = No |  |
|  | 1 = Yes |  |
|  |  |  |
| **4. HIV RISK PERCEPTION** | | |
| Next we will talk about how much you feel at risk of HIV. | | |
|  |  |  |
| 4.1. | How much do you personally feel at risk of HIV? | \|___\| |
|  | 1 = Not at all |  |
|  | 2 = A little |  |
|  | 3 = A lot |  |
|  |  |  |
|  |  |  |
|  |  |  |
| 4.2. | How much do you worry that your **own behaviours** put you at risk of HIV? | \|___\| |
|  | 1 = Not at all |  |
|  | 2 = A little |  |
|  | 3 = A lot |  |
|  |  |  |
| 4.3. | How much do you worry that **your partner or partners' behaviours** put you at risk of HIV? | \|___\| |
|  | 1 = Not at all |  |
|  | 2 = A little |  |
|  | 3 = A lot |  |
|  |  |  |
| **5. RISK REDUCTION BEHAVIOURS** | | |
| Next I will ask you about some of your sex behaviours. | | |
|  |  |  |
| 5.1. | When was the last time you came for a study visit? |  |
|  |  |  |
|  | _________________________________________________________________ |  |
|  |  |  |
| 5.2. | How many times have you had vaginal sex in the last three months [OR: *since the last time you came for a study visit*]? | \|___\| |
|  | 0 = Never |  |
|  | 1 = Once |  |
|  | 2 = 2-10 times |  |
|  | 3 = More than 10 times |  |
|  |  |  |
|  | *[IF 2.2 OR 2.3 = 1]* |  |
| 5.3. | How many times have you had anal sex in the last three months [OR: *since the last time you came for a study visit*]? | \|___\| |
|  | 0 = Never |  |
|  | 1 = Once |  |
|  | 2 = 2-10 times |  |
|  | 3 = More than 10 times |  |
|  |  |  |
| 5.4. | When was the last time you had vaginal sex? |  |
|  | *Note: Use EITHER whole days, whole weeks or whole months.* |  |
|  | 5.1.a. Number of DAYS ago | \|___\|___\| |
|  | 5.1.b. Number of WEEKS ago | \|___\|___\| |
|  | 5.1.c. Number of MONTHS ago | \|___\|___\| |
|  |  |  |
|  | *[IF 2.2 OR 2.3 = 1]* |  |
| 5.5. | When was the last time you had anal sex? |  |
|  | *Note: Use EITHER whole days, whole weeks or whole months.* |  |
|  | 5.2.a. Number of DAYS ago | \|___\|___\| |
|  | 5.2.b. Number of WEEKS ago | \|___\|___\| |
|  | 5.2.c. Number of MONTHS ago | \|___\|___\| |
|  |  |  |
| 5.6. | How would you describe the last man you had vaginal sex with? | \|___\| |
|  | 0 = Husband |  |
|  | 1 = Man I live with but am not married to |  |
|  | 2 = Regular partner |  |
|  | 3 = Other partner |  |
|  | 4 = New partner |  |
|  | 5 = Client |  |
|  | 6 = Other, specify: _________________________________________________ |  |
|  |  |  |
|  | *[IF 2.2 OR 2.3 = 1]* |  |
| 5.7. | How would you describe the last man you had anal sex with? | \|___\| |
|  | 0 = Husband |  |
|  | 1 = Man I live with but am not married to |  |
|  | 2 = Regular partner |  |
|  | 3 = Other partner |  |
|  | 4 = New partner |  |
|  | 5 = Client |  |
|  | 6 = Other, specify: _________________________________________________ |  |
|  |  |  |
| 5.8. | The last time you had sex, did you discuss condom use with your sexual partner? | \|___\| |
|  | 0 = No |  |
|  | 1 = Yes |  |
|  |  |  |
| 5.9. | The last time you had vaginal sex, did you use a male or female condom? | \|___\| |
|  | 0 = No |  |
|  | 1 = Yes, female condom |  |
|  | 2 = Yes, male condom |  |
|  |  |  |
|  | *[IF 2.2 OR 2.3 = 1]* |  |
| 5.10. | The last time you had anal sex, did you use a male or female condom? | \|___\| |
|  | 0 = No |  |
|  | 1 = Yes, female condom |  |
|  | 2 = Yes, male condom |  |
|  |  |  |
| 5.11. | The last time you had sex, how willing were you to have sex? Would you say you… | \|___\| |
|  | 1 = Were willing/wanted to have sex |  |
|  | 2 = Were persuaded/coerced *(i.e., did not want to have sex, were unsure, were talked into it, or had sex out of fear of being abandoned otherwise)* |  |
|  | 3 = Were physically forced/raped *(i.e., held down, hit or threatened with violence)* |  |
|  | 4 = Had sex for material things, money or drugs |  |
|  |  |  |
|  |  |  |
|  |  |  |
| 5.12. | The last time you had sex, did you or your partner use alcohol and/or drugs? | \|___\| |
|  | 1 = Yes, **I** used alcohol/drugs |  |
|  | 2 = Yes, **my partner** used alcohol/drugs |  |
|  | 3 = Yes, **both my partner and I** used alcohol/drugs |  |
|  | 4 = No, **neither** I nor my partner used alcohol/drugs |  |
|  |  |  |
| **6. STUDY PRODUCT USE** | | |
| Now we will talk about your experience using the [study product]. | | |
| *Note: [Insert name of study product when appropriate throughout.]* | | |
|  |  |  |
| 6.1. | *[IF A DAILY PRODUCT USER]* |  |
|  | Since your last visit, how often did you use the study product? | \|___\| |
|  | 0 = Never |  |
|  | 1 = Less than half the time |  |
|  | 2 = More than half the time |  |
|  | 3 = Always |  |
|  |  |  |
|  | *[IF A PERICOITAL GEL USER]* |  |
|  | Since your last visit, how many times did you use the pericoital gel? | \|___\| |
|  |  |  |
|  | *[IF A VAGINAL RING USER]* |  |
|  | Since your last visit, how many times did you remove the vaginal ring? | \|___\| |
|  |  |  |
| 6.2. | How easy or difficult is the [study product] to use? | \|___\| |
|  | *Note: Read responses aloud.* |  |
|  | 1 = Very easy |  |
|  | 2 = A little easy |  |
|  | 3 = A little difficult |  |
|  | 4 = Very difficult |  |
|  |  |  |
|  |  |  |
|  |  |  |
|  |  |  |
|  |  |  |
|  |  |  |
| 6.3. | Participants may not always use their [study product] as directed for many reasons. I will read a list of possible reasons some participants may have missed [taking a pill/applying their gel/using the vaginal ring]. Were any of the following a reason why you DID NOT use the [study product]? |  |
|  | *Note: Read all responses aloud. Mark all that apply with a 1.* |  |
|  | 6.3.a. [Never missed a dose/never removed the ring] | \|___\| |
|  | 6.3.b. Was away from home and left the [study product] behind | \|___\| |
|  | 6.3.c. Forgot/was unable to [take/apply/use] | \|___\| |
|  | 6.3.d. Did not want others to see you [take/apply/use] | \|___\| |
|  | 6.3.e. Worried partner would feel [study product] during sex | \|___\| |
|  | 6.3.f. Worried about side effects | \|___\| |
|  | 6.3.g. Felt sad or overwhelmed | \|___\| |
|  | 6.3.h. Ran out of [study product] | \|___\| |
|  | 6.3.i. Misplaced [study product] | \|___\| |
|  | 6.3.j. Shared [study product] with others | \|___\| |
|  | 6.3.k. Had too much alcohol | \|___\| |
|  | 6.3.l Other, specify: ________________________________________________ | \|___\| |
|  |  |  |
| 6.4. | How frequently did you use a male or female condom while using your study product? | \|___\| |
|  | 0 = Never |  |
|  | 1 = Less than half the time |  |
|  | 2 = More than half the time |  |
|  | 3 = Always |  |
|  |  |  |
| **7. ACCEPTABILITY** | | |
| Now I will ask you some questions about how you felt about using the [study product]. | | |
|  |  |  |
| 7.1. | In this clinical trial, how much did you like or dislike using the [study product]? | \|___\| |
|  | 1 = Disliked A LOT |  |
|  | 2 = Disliked A LITTLE |  |
|  | 3 = Liked A LITTLE |  |
|  | 4 = Liked A LOT |  |
|  |  |  |
|  |  |  |
|  |  |  |
|  |  |  |
|  |  |  |
| 7.2. | Imagine that the [study product] you were provided actually gave some protection against HIV or other STIs. If this were true, what would you like about it? |  |
|  | *Note: Read responses aloud, STARTING WITH "b". Mark all that apply with a 1.* |  |
|  | *7.2.a. Nothing* | \|___\| |
|  | 7.2.b. Some protection against HIV | \|___\| |
|  | 7.2.c. Some protection against STIs | \|___\| |
|  | 7.2.d. Can be used discreetly, without partner's knowledge | \|___\| |
|  | 7.2.e. Easier to use than other methods | \|___\| |
|  | 7.2.f. Method under my control/*[FOR INJECTABLE]* administered by a healthcare provider | \|___\| |
|  | 7.2.g. Does not interrupt sex | \|___\| |
|  | 7.2.h. Appearance and/or smell | \|___\| |
|  | 7.2.i Other, specify: ________________________________________________ | \|___\| |
|  |  |  |
| 7.3. | If the [study product] you were provided actually gave some protection against HIV or other STIs, what would you dislike about it? |  |
|  | *Note: Read responses aloud, STARTING WITH "b". Mark all that apply with a 1.* |  |
|  | *7.3.a. Nothing* | \|___\| |
|  | 7.3.b. May not protect fully against HIV | \|___\| |
|  | 7.3.c. Cannot be used discreetly, without partner's knowledge | \|___\| |
|  | 7.3.d. May cause harmful side effects | \|___\| |
|  | 7.3.e. May be painful | \|___\| |
|  | 7.3.f. *[FOR PERICOITAL GEL]* Interrupts sex | \|___\| |
|  | 7.3.g. *[FOR DAILY PRODUCT/PERICOITAL GEL/VAGINAL RING]* Difficult to store or discard | \|___\| |
|  | 7.3.h. *[FOR DAILY PRODUCT/PERICOITAL GEL/VAGINAL RING]* Remembering to use it | \|___\| |
|  | 7.3.i. *[FOR DAILY PRODUCT/PERICOITAL GEL/VAGINAL RING]* Taste or feel of [study product] | \|___\| |
|  | 7.3.j. *[FOR INJECTABLE]* Must be administered by a healthcare provider | \|___\| |
|  | 7.3.k. Other, specify: _______________________________________________ | \|___\| |
|  |  |  |
| 7.4. | If it were possible to change the way the [study product] was given/taken, what kind of changes would you recommend? |  |
|  | *Note: DO NOT read responses aloud. Mark all that apply.* |  |
|  | 7.4.a. None | \|___\| |
|  | 7.4.b. Reduce the [size of ring or pill/volume of gel or liquid] | \|___\| |
|  | 7.4.c. Increase the duration of protection offered by the [study product] | \|___\| |
|  | 7.4.d. *[FOR INJECTABLE]* Have only one injection with a larger dose | \|___\| |
|  | 7.4.e. *[FOR INJECTABLE]* Have only one injection with a smaller dose | \|___\| |
|  | 7.4.f. *[FOR INJECTABLE]* Receive the injection in the arm, instead of the buttock | \|___\| |
|  | 7.4.g. Other, specify: _______________________________________________ | \|___\| |
|  |  |  |
|  |  |  |
| 7.5. | If you wanted to protect yourself from getting HIV, which product would you prefer to use? | \|___\| |
|  | 0 = Prefer not using a product |  |
|  | 1 = Condoms only |  |
|  | 2 = [Study product] only |  |
|  | 3 = Condoms and [study product] together |  |
|  |  |  |
| 7.6. | If you could receive the same level of protection (from pregnancy or disease) by taking any one of the following products, which one would you prefer? | \|___\| |
|  | *Note: Read each category aloud. Select one.* |  |
|  | 0 = Does not matter |  |
|  | 1 = Male condoms |  |
|  | 2 = Female condoms |  |
|  | 3 = Oral pill taken every day |  |
|  | 4 = Injection received once every 2 months |  |
|  | 5 = Vaginal ring (flexible medicine-filled ring inserted in the vagina, changed once every month) |  |
|  | 6 = Vaginal gel (inserted in the vagina with an applicator before and/or after sex) |  |
|  | 7 = Other, specify: _________________________________________________ |  |
|  |  |  |
| 7.7. | Would you tell your partner(s) about your use of the product you selected? | \|___\| |
|  | 0 = No |  |
|  | 1 = Yes |  |
|  |  |  |
| **8. CLINICAL TRIAL PARTICIPATION** | | |
| Finally, we I will ask you some questions about your experience participating in this trial. | | |
|  |  |  |
| 8.1. | During the time you participated in this clinical trial, did you feel like you could honestly provide information about your sexual behaviour to the clinic staff? | \|___\| |
|  | 0 = No |  |
|  | 1 = Yes |  |
|  |  |  |
| 8.2. | Did you ever feel that people looked at you different because you were using the [study product]? |  |
|  | 0 = No |  |
|  | 1 = Yes |  |
|  |  |  |
| 8.3. | Did you tell anyone else that you were using this product? | \|___\| |
|  | 0 = No *[SKIP TO 8.5.]* |  |
|  | 1 = Yes |  |
|  |  |  |
|  |  |  |
|  |  |  |
|  |  |  |
|  |  |  |
|  | *[IF 8.3 = 1]* |  |
| 8.4. | Who did you tell? |  |
|  | *Note: Mark all that apply with a 1.* |  |
|  | 8.4.a. Parents | \|___\| |
|  | 8.4.b. Sex partner | \|___\| |
|  | 8.4.c. Friends | \|___\| |
|  | 8.4.d. Other, specify: _______________________________________________ | \|___\| |
|  |  |  |
| 8.5. | Did you experience any conflict with your parents, sex partner, or friends because you were using this product? |  |
|  | 0 = No *[SKIP TO END.]* |  |
|  | 1 = Yes |  |
|  | *[IF 8.5 = 1]* |  |
| 8.6. | Whom did you experience conflict with? |  |
|  | *Note: Mark all that apply with a 1.* |  |
|  | 8.6.a. Parents | \|___\| |
|  | 8.6.b. Sex partner | \|___\| |
|  | 8.6.c. Friends | \|___\| |
|  | 8.6.d. Other, specify: _______________________________________________ | \|___\| |
|  |  |  |
|  | We are now finished with all of the questions. Thank you for taking the time to answer them. Do you have any questions for me? |  |
